# Respondent-driven sampling for identification of HIV- and HCV-infected people who inject drugs and men who have sex with men in India: A cross-sectional, community-based analysis

**DOI:** 10.1371/journal.pmed.1002460

**Published:** 2017-11-28

**Authors:** Sunil S. Solomon, Allison M. McFall, Gregory M. Lucas, Aylur K. Srikrishnan, Muniratnam S. Kumar, Santhanam Anand, Thomas C. Quinn, David D. Celentano, Shruti H. Mehta

**Affiliations:** 1 Johns Hopkins University School of Medicine, Baltimore, Maryland, United States of America; 2 Johns Hopkins Bloomberg School of Public Health, Baltimore, Maryland, United States of America; 3 Y.R. Gaitonde Centre for AIDS Research and Education, Chennai, India; 4 Division of Intramural Research, National Institute of Allergy and Infectious Diseases, National Institutes of Health, Bethesda, Maryland, United States of America; University of Melbourne, AUSTRALIA

## Abstract

**Background:**

A major barrier to achieving ambitious targets for global control of HIV and hepatitis C virus (HCV) is low levels of awareness of infection among key populations such as men who have sex with men (MSM) and people who inject drugs (PWID). We explored the potential of a strategy routinely used for surveillance in these groups, respondent-driven sampling (RDS), to be used as an intervention to identify HIV- and HCV-infected PWID and MSM who are unaware of their status and those who are viremic across 26 Indian cities at various epidemic stages.

**Methods and findings:**

Data were collected as part of the baseline assessment of an ongoing cluster-randomized trial. RDS was used to accrue participants at 27 sites (15 PWID sites and 12 MSM sites) selected to reflect varying stages of the HIV epidemic among MSM and PWID in India. A total of 56 seeds recruited a sample of 26,447 persons (approximately 1,000 participants per site) between October 1, 2012, and December 19, 2013. Across MSM sites (*n =* 11,997), the median age was 25 years and the median number of lifetime male partners was 8. Across PWID sites (*n =* 14,450), 92.4% were male, the median age was 30 years, and 87.5% reported injection in the prior 6 months. RDS identified 4,051 HIV-infected persons, of whom 2,325 (57.4%) were unaware of their HIV infection and 2,816 (69.5%) were HIV viremic. It also identified 5,777 HCV-infected persons, of whom 5,337 (92.4%) were unaware that they were infected with HCV and 4,728 (81.8%) were viremic. In the overall sample (both MSM and PWID), the prevalence of HIV-infected persons who were unaware of their status increased with sampling depth, from 7.9% in participants recruited in waves 1 through 5 to 12.8% among those recruited in waves 26 and above (*p*-value for trend < 0.001). The overall detection rate of people unaware of their HIV infection was 0.5 persons per day, and the detection rate of HIV-infected persons with viremia (regardless of their awareness status) was 0.7 per day. The detection rate of HIV viremic individuals was positively associated with underlying HIV prevalence and the prevalence of HIV viremia (linear regression coefficient per 1-percentage-point increase in prevalence: 0.05 and 0.07, respectively). The median detection rate of PWID who were unaware of their HCV infection was 2.5 per day. The cost of identifying 1 unaware HIV-infected individual ranged from US$51 to US$2,072 across PWID sites and from US$189 to US$5,367 across MSM sites. The mean additional cost of identifying 1 unaware HCV-infected PWID was US$13 (site range: US$7–US$140). Limitations of the study include the exclusivity of study sites to India, lack of prior HIV/HCV diagnosis confirmation with clinic records, and lack of cost data from other case-finding approaches commonly used in India.

**Conclusions:**

In this study, RDS was able to rapidly identify at nominal cost a substantial number of unaware and viremic HIV-infected and HCV-infected individuals who were currently not being reached by existing programs and who were at high risk for transmission. Combining RDS (or other network-driven recruitment approaches) with strategies focused on linkage to care, particularly in high-burden settings, may be a viable option for achieving the 90-90-90 targets in key populations in resource-limited settings.

## Abstract 2

In a large cross-sectional study done in India, Sunil Solomon and colleagues report on respondent-driven sampling for identification of HIV and HCV infections.

## Author summary

**Why was this study done?:**

- Men who have sex with men (MSM) and people who inject drugs (PWID) bear a high burden of HIV and HCV, and fall behind other at-risk groups in achieving Joint United Nations Programme on HIV/AIDS (UNAIDS) 90-90-90 targets.
- The behaviors of PWID and MSM (injection drug use and engagement in same-sex relationships) are highly stigmatized in many low- and middle-income countries, resulting in large segments of these populations being hidden.
- Finding PWID and MSM who are HIV-infected but unaware of their status and not in treatment is critical to curbing the HIV epidemic.

**What did the researchers do and find?:**

- We used data collected as part of an ongoing cluster-randomized trial to explore the potential of respondent-driven sampling (RDS), a chain-referral recruitment strategy, to be used as an intervention to identify unaware and viremic HIV-infected MSM and PWID across 26 Indian cities. We further evaluated the potential of incorporating hepatitis C virus (HCV) testing for PWID.
- RDS was effective in identifying unaware and viremic HIV-infected MSM and PWID, as well as unaware and viremic HCV-infected PWID.
- The longer RDS ran (more waves), the more likely it was to identify unaware and viremic HIV-infected MSM and PWID.
- RDS was most effective in settings with high HIV and HCV burden and low service availability.
- Incorporating HCV testing into HIV programs for PWID is a cost-effective way of identifying PWID unaware of their HCV infection.

**What do these findings mean?:**

- As the HIV community embraces the UNAIDS 90-90-90 targets, respondent-driven recruitment could be a critical tool in achieving the first 90 target among MSM and PWID in low-and-middle-income countries.
- Programs targeting drug-using populations should consider combining HIV and HCV screening for maximal benefit to the population.

## Introduction

Effective treatment for HIV and hepatitis C virus (HCV) infection has generated optimism that epidemic control and even elimination may be possible with high treatment coverage rates [1–3]. For HIV, the advent of highly active antiretroviral therapy (HAART) and subsequent therapeutics that are more potent, less cumbersome, and less toxic have transformed HIV from an invariably fatal disease into a chronic, manageable condition [4]. For HCV, while therapeutic developments have come more recently, they have been equally remarkable. New direct antiviral agents have ushered in an era where 95% of those treated can be cured with 8–12 weeks of therapy [5–7]. With these developments for both HIV and HCV, the scientific community has recognized the treatment benefits, both for the individual and the population, of “treatment as prevention.” Accordingly, the Joint United Nations Programme on HIV/AIDS (UNAIDS) has set targets such as “zero new HIV infections” and, more recently, the 90-90-90 targets, which set 90% benchmarks for diagnosis of infected persons, consistent HAART use in diagnosed persons, and viral suppression in treated persons [3]. For HCV, even though therapeutic developments have been more recent, conversations have already turned towards eradication, given the availability of curative therapy [2,8].

However, both HIV and HCV, which together affect 100–120 million people globally and account for more than 1.5 million deaths each year, face similar challenges in meeting these ambitious targets [9,10]. Moreover, programs for both will need to reach vulnerable populations such as people who inject drugs (PWID) and men who have sex with men (MSM), who bear a disproportionately high burden of these infections in low- and middle-income countries (LMICs). In such settings, the greatest barrier to achieving these ambitious targets is low levels of awareness of infection—more than 50% of MSM and PWID in LMICs are unaware of their HIV status [11], and as many as 90% of PWID in LMICs are unaware of their HCV status or aware but not linked to care [12]. It is thus critical to identify interventions to close this gap.

MSM and PWID have several characteristics in common including, in some settings, criminalization. Injection drug use is universally illegal, and there are laws that criminalize homosexuality (anti-sodomy laws) in more than 70 countries, including 33 countries in Africa and 25 in Asia. As a consequence, both MSM and PWID experience high levels of stigma in most LMICs, which often drives them further underground. Yet both MSM and PWID have strong social ties, leading to high levels of interconnectedness that have previously been leveraged for public health research and surveillance. For example, a key sampling strategy for measuring disease burden in MSM and PWID, called respondent-driven sampling (RDS), relies on individuals to recruit their peers through a reciprocal incentive system [13,14]. While RDS was in some ways initially conceived as a means for achieving HIV prevention [15,16], it has primarily been utilized as a tool for surveillance, and its potential to identify infected individuals and engage them in care has been insufficiently evaluated.

Accordingly, we explored the potential for RDS to be used as an intervention to identify HIV- and HCV-infected PWID and MSM who are unaware of their infection status or who are aware but not engaged in optimal care (detectable viremia) across 26 Indian cities at various epidemic stages. Specifically, using RDS, we examined the rate of, and the factors associated with, identification of people unaware of their infection status or aware of their infection status but with incomplete viral suppression, as well as associated costs.

## Methods

### Ethical clearances

All participants were required to provide verbal informed consent to be included in this study. This study was approved by the institutional review boards of the Johns Hopkins University Schools of Medicine and Public Health and the Y.R. Gaitonde Centre for AIDS Research and Education.

### Study setting

Data used in these analyses were collected as part of the baseline assessment of an ongoing cluster-randomized trial among MSM and PWID in India, Integrated Care Centers to Improve HIV Outcomes in Vulnerable Indian Populations: National Collaboration on AIDS (NCA study) (ClinicalTrials.gov identifier: NCT01686750) [17]. RDS was used to accrue participants for the baseline assessment in order to characterize HIV and HCV (PWID sites only) epidemiology among these key populations in candidate cities for the trial (*n =* 27 sites [15 PWID and 12 MSM] across 26 Indian cities; New Delhi had both an MSM and a PWID site). Recruitment took place between October 1, 2012, and December 19, 2013. Sites were selected to reflect varying stages of the HIV epidemic among MSM and PWID in India. One RDS field site was established in each city. The sites were on average about 1,000 square feet and usually comprised 6 soundproof rooms (1 for the site coordinator, 1 for the counselor, and 4 for the interviewers). Additionally, there was a laboratory and a phlebotomist at each site, and a logistics assistant to guide participants within the sites.

### Study population

The study population was accrued using RDS, a chain-referral recruitment strategy that is effective in recruiting hard-to-reach populations [13,14]. At each site, prior to the RDS, ethnography was conducted to identify seeds—individuals considered influential in the local (i.e., city) MSM/PWID community who served as the starting point for RDS. Each seed was given 2 hologram-labeled referral coupons to recruit up to 2 network members (i.e., others living in their community that they know inject drugs or have sex with men). Unique identification numbers on the coupons established the recruiter–recruit relationships. Though RDS studies have previously provided up to 5 coupons, we limited to 2 coupons since RDS theory assumes that as the RDS penetrates deeper into a community (i.e., more waves), the bias introduced by ad hoc seed selection is diminished, resulting potentially in a more representative sample. The network members returned to the site with the coupon and, if eligible, were enrolled and asked to complete study assessments. These recruited individuals were considered wave 1 of recruitment and were each given 2 referral coupons to recruit up to 2 members of their network. The next round of individuals recruited and enrolled were considered wave 2, and so on. Recruitment continued at each site until 1,000 eligible participants (prespecified sample size) had been sampled. Two seeds (started simultaneously) were used at all but 1 MSM site (New Delhi) and 1 PWID site (Gangtok), where a third seed was included. The third seed was initiated since recruitment in these 2 cities was relatively slower than in other cities included in this sample. Participants were reimbursed both for participation (INR 250 [US$3.8]) and for each eligible study participant they referred to the study (INR 50 [US$0.8]). Participants provided a fingerprint, which generated a unique hexadecimal code to prevent duplicate enrollment. Additional details on the recruitment process are included in S1 Text.

Eligibility criteria for both MSM and PWID included (1) age ≥ 18 years, (2) provision of informed consent, and (3) possession of a valid RDS referral coupon. In addition to these criteria, MSM participants also had to self-identify as male (transgender/hijras were excluded) and report sex with a man in the prior 12 months. Similarly, PWID participants had to report drug injection in the prior 24 months. There were no exclusions based on sex at PWID sites. Transgender individuals who visited a PWID site with a valid referral coupon were included in the study.

### Study procedures

Participants completed an interviewer-administered electronic survey that captured information on demographics, risk behaviors, and access to HIV and HCV diagnostic and treatment services. Upon completion of the survey, participants provided a venous sample following HIV pre-test counseling. Rapid HIV testing was performed using 3 rapid tests: Alere Determine HIV-1/2 (Alere Medical, Chiba, Japan), First Response HIV Card Test 1–2.0 (Premier Medical Corporation, Daman, India), and Signal Flow Through HIV 1+2 Spot/Immunodot Test Kit (Span Diagnostics, Surat, India). HIV results were delivered to participants on the same day with appropriate post-test counseling. All samples were shipped to the central laboratory in Chennai, India, for further testing and storage. Absolute CD4+ count results were made available to HIV-infected study participants within 14 days of their study visit. HIV-1 RNA was quantified in HIV-positive participants using a RealTime HIV-1 assay (Abbott Laboratories, Abbott Park, Illinois, US) with a lower limit of quantification of 150 copies/ml. Among participants from PWID sites, HCV antibody testing was performed on stored specimens in 2014 using the Genedia HCV ELISA 3.0 (Green Cross Medical Science, Chungbuk, Korea), and HCV RNA was quantified using the Abbott RealTime HCV assay (Abbott Laboratories, Abbott Park, Illinois, US).

### Statistical methods

The analyses presented in this report were not prespecified as part of the study protocol for the cluster-randomized trial from which these data derive, and a prospective analytical plan was not prepared. The primary objective of this analysis was to assess the utility of RDS as an intervention to identify out-of-care (viremic) individuals in key populations across India. As treatment targets for both HIV and HCV are evolving, we considered several definitions of target groups to enhance applicability across settings—Group 1: HIV-infected persons unaware of their status (based on self-report); Group 2: HIV-infected persons with HIV viremia regardless of their awareness or care status; Group 3: HCV-infected PWID unaware of their status (based on self-report); and Group 4: HCV-infected PWID with HCV viremia regardless of their awareness status. Group 1 addresses the first UNAIDS 90-90-90 target, while Group 2 addresses the third target and is relevant to the evolving consensus that viral suppression in a high proportion of infected persons can slow or stop the epidemic [3]. HIV viremia was defined as HIV RNA greater than 150 copies/ml in an HIV-infected person regardless of awareness or care status. HCV viremia was defined as HCV RNA greater than 30 IU/ml in an HCV-infected person regardless of awareness, representing persons with active HCV infection.

We explored several outcomes that we hypothesized could impact the utility of an RDS recruitment approach to help achieve critical HIV and HCV program targets. First, we examined the association between depth of RDS (i.e., recruitment wave) and prevalence of viremic/unaware individuals using line graphs and logistic regression. Second, we estimated the rate at which RDS identified all 4 target groups; this rate was calculated by dividing the number of unaware/viremic individuals identified in a city by the number of days it took to complete the RDS in the city, resulting in a rate of detection per day. Linear regression was used to explore community-level characteristics associated with detection rates across the 27 sites. Community-level characteristics such as HIV/HCV prevalence were calculated using the RDS-II estimator, which weights values using the inverse of network size (i.e., number of MSM/PWID seen in the prior 30 days). Third, we estimated the cost per unaware/viremic individual identified. The cost per individual was calculated by dividing the total cost to complete RDS in a city (e.g., site staffing, rent, utilities, incentives and compensation for RDS, site communication costs), after subtracting research-related expenses (e.g., sample shipping for storage, assays for diseases not related to the outcomes of interest such as HSV-2 and syphilis, investigator costs), by the number of unaware/viremic individuals identified in a city. For estimation of costs, we assumed US$1 was equal to 65 Indian rupees (INR) and utilized market value for tests: HIV antibody, INR 200 (US$3.1); HIV RNA, INR 4,400 (US$67.7); HCV antibody, INR 300 (US$4.6); and HCV RNA, INR 6,500 (US$100.0). Seeds were excluded from all analyses. There were minimal missing data; therefore, we used a complete case analysis approach (observations with missing data were dropped); 10 HIV-infected individuals did not have HIV RNA measurement, and 4 HCV-infected PWID did not have HCV RNA measurement. Pin (postal) code of residence by wave was mapped using ArcGIS version 10.2 (Redlands, CA, US). All statistical analyses were performed using Stata version 13 (StataCorp, College Station, TX, US).

## Results

### Respondent-driven sampling and population summary metrics

**Fig 1:**
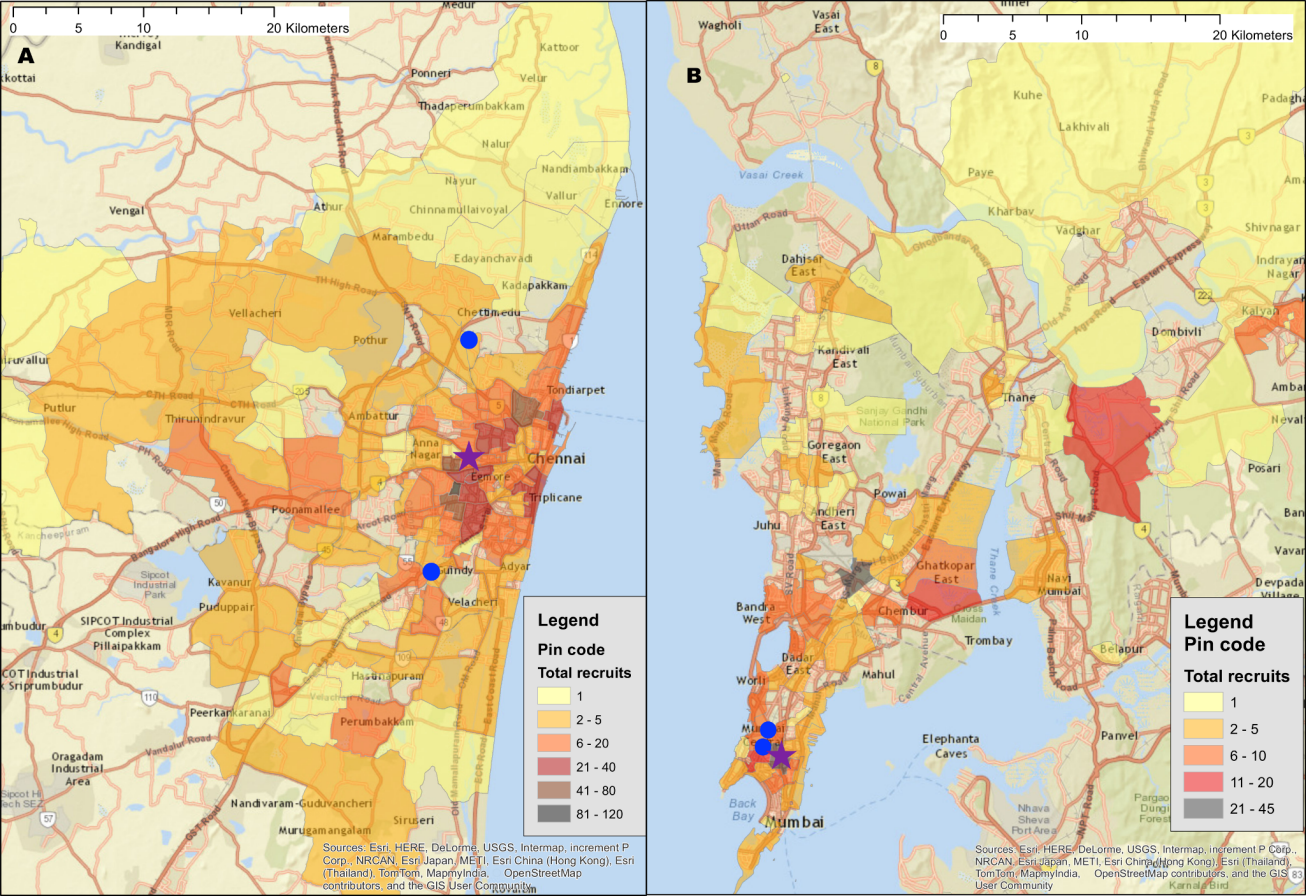
Recruitment of participants across a city using respondent-driven sampling. (A) Heatmap of the recruitment of men who have sex with men in Chennai by pin (postal) code of residence. (B) Heatmap of the recruitment of people who inject drugs in Mumbai by pin code of residence. The purple star marks the respondent-driven sampling field site, and the blue circles indicate the pin codes of the 2 seeds. Pin code data were not available for all participants. Map generated using OpenStreetMap (OpenStreetMap contributors, under the Open Database License, for which terms are available at http://opendatacommons.org/licenses/odbl/1.0/).

A total of 56 seeds led to the recruitment of 26,447 (*n =* 11,997 MSM; *n =* 14,450 PWID) persons at 27 sites distributed across 26 Indian cities in 15 states (Table 1) (S1 Fig depicts the flow diagram of the study population). Recruitment of MSM was initiated on October 1, 2012, and ended June 24, 2013; recruitment of PWID was initiated on January 1, 2013, and ended December 19, 2013. Approximately 1,000 participants were recruited per site, with the exception of Moreh (in Manipur), where RDS had to be terminated due to civil unrest. In each city, only 1 RDS field site was established (except New Delhi, which had both an MSM and a PWID site). Despite this, participants were recruited across large distances, some of which require travel times above 2 hours (Fig 1). The site median time to recruit MSM and PWID samples was 99 and 135 days, respectively, ranging from 52 days in Churachandpur (in Manipur; PWID sample) to 200 days in Chandigarh (Union Territory; PWID sample). The median depth of the MSM and PWID samples was 21 and 22 waves, respectively. At MSM sites, the median age was 25 years and the median number of lifetime male partners was 8. Across PWID sites, 92.4% were male, the median age was 30 years, and 87.5% reported injection in the prior 6 months. The median site-level HIV prevalence was 13.0% (range: 2.0%–43.3%) (Table 1), and the median site-level HCV antibody prevalence across PWID sites was 45.5% (range: 4.1%–68.4%). Other characteristics of these samples and factors associated with HIV incidence and prevalence have been described elsewhere [18,19].

**Table 1:** Summary respondent-driven sampling recruitment metrics and HIV/HCV characteristics.

Metric/characteristic	Overall (27 sites)	MSM sites only (12 sites)	PWID sites only (15 sites)
Total number recruited (seeds)	26,447 (56)	11,997 (25)	14,450 (31)
Median recruitment time in days (range)	112 (52–200)	99 (70–157)	135 (52–200)
Median number of waves (range)	21 (11–50)	21 (11–28)	22 (12–50)
Median site-level HIV prevalence (%) (range)	13.0 (2.0–43.3)	8.6 (2.0–18.8)	19.8 (6.1–43.3)
Weighted median site-level HIV prevalence (%) (range)*	11.2 (1.7–44.9)	6.4 (1.7–13.1)	18.1 (6.0–44.9)
Median site-level proportion of HIV-positive participants unaware of status (%) (range)	61.1 (7.6–100)	63.6 (7.6–100)	56.7 (7.8–97.2)
Weighted median site-level proportion of HIV-positive participants unaware of status (%) (range)*	64.1 (7.2–100)	70.0 (10.1–100)	59.2 (7.2–97.6)
Median site-level prevalence of HIV viremia (%) (range)	9.0 (1.8–33.8)	5.0 (1.8–13.2)	14.2 (2.8–33.8)
Weighted median site-level prevalence of HIV viremia (%) (range)*	7.5 (0.8–29.7)	3.6 (0.8–10.1)	11.4 (2.7–29.7)
Median site-level HCV prevalence (%) (range)	—	—	45.5 (4.1–68.4)
Weighted median site-level HCV prevalence (%) (range)*	—	—	41.1 (4.9–64.9)
Median site-level proportion of HCV-positive participants unaware of status (%) (range)	—	—	95.4 (75.6–99.7)
Weighted median site-level proportion of HCV-positive participants unaware of status (%) (range)*	—	—	94.4 (70.2–99.9)
Median site-level prevalence of HCV viremia (%) (range)	—	—	37.0 (3.5–58.0)
Weighted median site-level prevalence of HCV viremia (%) (range)*	—	—	34.1 (4.5–53.9)

*Weighted using RDS-II estimator.

HCV, hepatitis C virus; MSM, men who have sex with men; PWID, people who inject drugs.

Of the 4,051 HIV-infected persons recruited, 2,325 (57.4%) reported that they were unaware of their HIV infection and 2,816 (69.5%) had HIV viremia. At PWID sites, of the 5,777 PWID positive for HCV antibodies, 5,337 (92.4%) were unaware that they were infected with HCV and 4,728 were HCV viremic (81.8%).

### Prevalence of unaware and viremic individuals by recruitment wave

**Fig 2:**
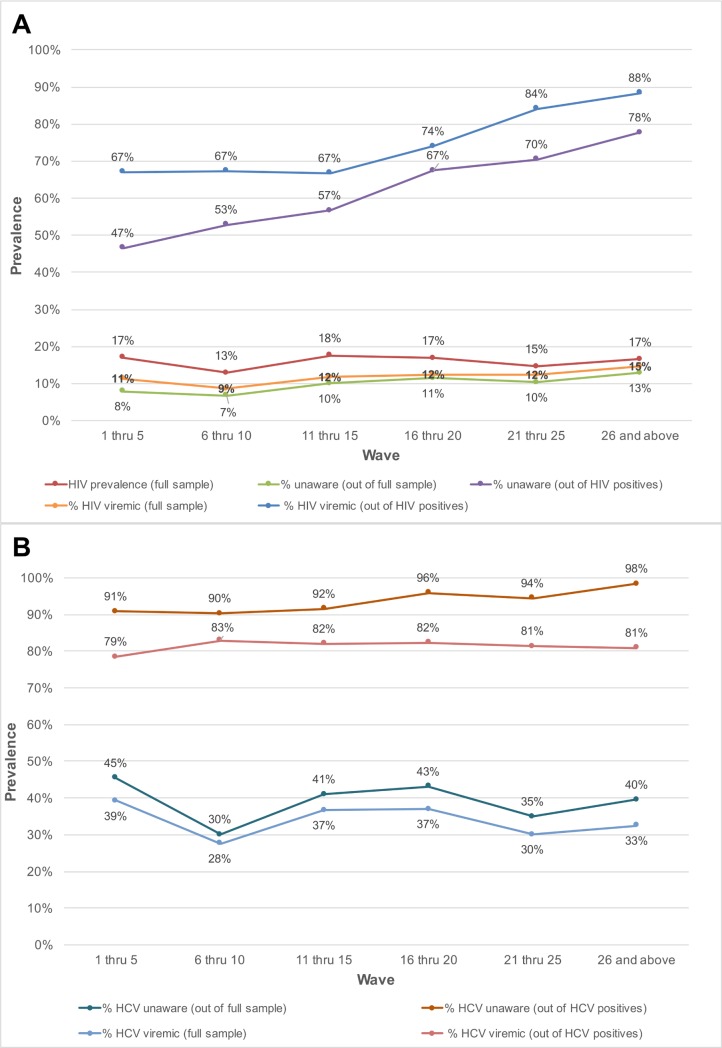
Prevalence of unaware and viremic individuals by respondent-driven sampling recruitment wave among PWID and MSM in India. (A) Prevalence of unaware HIV-infected and HIV viremic participants by recruitment wave pooled across the MSM and PWID samples (27 sites). (B) Prevalence of unaware HCV-infected and HCV viremic participants by recruitment wave across the 15 PWID sites. HCV, hepatitis C virus; MSM, men who have sex with men; PWID, people who inject drugs.

The prevalence of HIV-infected persons who were unaware of their status in the overall sample increased with sampling depth, from 7.9% in participants recruited in waves 1 through 5 to 12.8% among those recruited in waves 26 and above (*p*-value for trend < 0.001; Table 2). Among HIV-positive persons, the proportion who were unaware of their HIV infection increased from 46.5% in waves 1–5 to 77.7% in waves 26 and above (*p*-value for trend < 0.001; Fig 2). The odds ratio of identifying persons unaware of their HIV infection increased significantly by recruitment wave (odds ratio per 5-recruitment-wave increase: 1.31; 95% CI: 1.24, 1.38; Table 2). The prevalence of HIV viremic individuals recruited increased from 11.3% in waves 1–5 to 14.6% in waves 26 and above. Among HIV-positive persons, the proportion with viremia increased from 67% to 88.4% between waves 1–5 and waves 26 and above; in this group, there was a 3.73 higher odds of identifying a viremic person in waves 26 and above compared to waves 1 through 5 (95% CI: 2.32, 6.01). With respect to HCV, the odds of detecting HCV-infected PWID who were unaware of their HCV infection was 1.32-fold higher (95% CI: 1.22, 1.44) per 5-recruitment-wave increase, but there was no association between recruitment depth and identification of HCV viremic PWID.

**Table 2:** Association of respondent-driven sampling depth and prevalence of unaware and viremic persons.

Sampling depth	Odds ratio (95% confidence interval)					
	Prevalence of unaware HIV+ (full sample; *n =* 26,444)	Prevalence of unaware HIV+ (HIV+ only; *n =* 4,051)	Prevalence of HIV viremic individuals (full sample; *n =* 26,444)	Prevalence of HIV viremic individuals (HIV+ only; *n =* 4,051)	Prevalence of unaware HCV+ (HCV+ PWID only; *n =* 5,777)	Prevalence of HCV viremic individuals (HCV+ PWID only; *n =* 5,777)
*Model 1 (wave modeled as a continuous variable)*						
**Per 5-wave increase**	1.18 (1.14, 1.22)	1.31 (1.24, 1.38)	1.12 (1.09, 1.16)	1.23 (1.16, 1.31)	1.32 (1.22, 1.44)	1.00 (0.96, 1.06)
*Model 2 (wave modeled as categorical variable)*						
**Wave of recruitment**						
1–5	1	1	1	1	1	1
6–10	0.85 (0.72, 1.01)	1.29 (1.03, 1.62)	0.74 (0.64, 0.85)	1.02 (0.90, 1.29)	0.94 (0.69, 1.29)	1.33 (1.06, 1.67)
11–15	1.29 (1.09, 1.53)	1.51 (1.20, 1.89)	1.04 (0.90, 1.20)	1.00 (0.79, 1.27)	1.09 (0.79, 1.51)	1.25 (0.99, 1.57)
16–20	1.50 (1.24, 1.81)	2.38 (1.81, 3.13)	1.11 (0.94, 1.32)	1.39 (1.04, 1.86)	2.37 (1.51, 3.72)	1.27 (0.97, 1.66)
21–25	1.34 (1.05, 1.70)	2.74 (1.87, 4.01)	1.10 (0.89, 1.36)	2.64 (1.67, 4.17)	1.71 (0.99, 2.97)	1.20 (0.85, 1.69)
26+	1.71 (1.37, 2.15)	4.00 (2.72, 5.87)	1.34 (1.09, 1.64)	3.79 (2.36. 6.10)	6.05 (2.86, 12.8)	1.16 (0.86, 1.56)

### Rate of detection of unaware and viremic individuals

The detection rate of HIV-infected individuals with viremia overall was 0.7 persons per day, with median detection rates across PWID and MSM sites of 0.7 and 0.4 per day, respectively. However, there was substantial variability, ranging from 1 unaware PWID identified every 10 days in Gangtok to 3.2 detected each day in Churachandpur (Table 3). Among the MSM sites, Hyderabad was the only site where the rate exceeded 1 per day (1.2 unaware HIV-infected MSM per day; Table 4). The detection rate of HIV-infected PWID with viremia ranged from 1 per 3.3 days in Gangtok and Lunglei to 2.7 per day in Churachandpur. The median detection rate of HIV viremic MSM was 0.5 per day (site range: 0.2–1.1 per day). The detection rate of HIV viremic individuals was positively associated with underlying community HIV prevalence and prevalence of HIV viremia (linear regression coefficient per 1-percentage-point increase in prevalence: 0.05 and 0.07, respectively).

**Table 3:** Respondent-driven sampling recruitment metrics and HIV/HCV characteristics across PWID sites.

Metric/characteristic	Northeast cities							North or central cities							
	AIZ	CCP	DIM	GTK	IMP	LGL	MOR	DEL	MUM	AMR	CDH	LUD	BBE	BIL	KAN
Total number recruited	1,000	1,000	1,000	1,000	1,000	1,000	457	999	999	999	996	1,000	1,000	1,000	1,000
Number of seeds	2	2	2	3	2	2	2	2	2	2	2	2	2	2	2
Recruitment time (days)	135	52	125	94	89	139	80	112	145	139	200	137	65	180	190
Number of waves (by seed)	21 (21, 18)	17 (16, 17)	14 (14, 14)	13 (12, 13, 10)	26 (26, 18)	22 (22, 12)	21 (17, 21)	24 (24, 9)	14 (14, 13)	31 (31, 22)	50 (50, 1)	38 (38, 16)	12 (12, 11)	38 (38, 1)	26 (25, 26)
Number of recruits by seed	749, 251	392, 608	707, 293	375, 460, 165	927, 73	714, 271^	136, 321	952, 47	415, 584	872, 127	994, 2	931, 69	592, 408	998, 2	732, 268
Coupon response rate (%)	50	67	50	50	56	50	50	50	50	50	51	50	50	50	58
Median network size*	20	20	7	7	15	20	20	12	10	20	10	15	4	10	10
Prevalence of unaware HIV-infected persons (%)	9.8	16.8	4.3	0.8	18.1	3.1	17.7	13.5	4.7	13.8	10.0	16.7	1.7	12.5	34.3
Rate of detection of unaware HIV-infected persons per day	0.73	3.23	0.34	0.09	2.03	0.22	1.03	1.21	0.32	0.99	0.50	1.22	0.26	0.69	1.81
Cost per unaware HIV-infected person identified (USD)	230.0	83.4	550.1	2,071.7	145.3	694.9	153.5	139.0	516.6	130.8	297.9	85.4	1,317.4	126.3	50.8
Prevalence of HIV viremia (%)	23.3	14.1	9.3	2.8	18.2	3.9	28.2	15.6	8.4	20.1	10.7	19.5	3.1	14.3	33.8
Rate of detection of HIV viremic persons per day	1.73	2.71	0.74	0.31	2.06	0.28	1.63	1.39	0.58	1.45	0.54	1.42	0.48	0.79	1.78
Cost per HIV viremic person identified (USD)	185.6	233.8	397.7	811.9	262.4	729.4	200.9	192.7	365.6	166.2	355.0	152.6	857.8	183.3	122.5
HCV prevalence (%)	68,4	59.1	10.5	4.2	68.5	15.8	45.5	45.7	34.9	52.6	54.8	34.2	8.4	33.4	67.7
Prevalence of unaware HCV-infected persons (%)	54.3	52.8	10.2	3.2	58.0	14.9	41.6	44.8	33.3	48.1	53.9	33.6	8.1	33.3	67.3
Rate of detection of unaware HCV-infected persons per day	4.03	10.2	0.82	0.34	6.53	1.07	2.39	4.0	2.30	3.47	2.69	2.46	1.25	1.86	3.55
Cost per unaware HCV-infected person identified (USD)	49.9	35.2	277.2	662.6	53.2	175.6	77.0	52.2	86.8	47.0	64.0	56.0	333.6	61.1	32.8
Prevalence of HCV viremia (%)	58.0	47.1	8.8	3.5	55.4	12.9	37.4	37.0	27.6	42.1	43.4	27.3	7.2	28.9	56.7
Rate of detection of HCV viremic persons per day	4.3	9.06	0.70	0.37	6.22	0.93	2.14	3.3	1.90	3.03	2.16	1.99	1.11	1.61	2.98
Cost per HCV viremic person identified (USD)	164.8	164.8	439.5	722.9	179.3	324.6	207.7	186.4	230.8	178.5	205.8	194.1	491.9	185.9	158.4

*Number of PWID seen in prior 30 days.

^Error in coupon tracking resulted in missing recruiter–recruit link for 3 study participants who recruited 12 subsequent recruits.

AIZ, Aizawl; AMR, Amritsar; BBE, Bhubaneswar; BIL, Bilaspur; CCP, Churachandpur; CDH, Chandigarh; DEL, New Delhi; DIM, Dimapur; GTK, Gangtok; HCV, hepatitis C virus; IMP, Imphal; KAN, Kanpur; LGL, Lunglei; LUD, Ludhiana; MOR, Moreh; MUM, Mumbai; PWID, people who inject drugs; USD, US dollars.

**Table 4:** Respondent-driven sampling recruitment metrics and HIV characteristics across MSM sites.

Metric/characteristic	Andhra Pradesh			Karnataka			Tamil Nadu			Central and north India		
	HYD	VJW	VZG	BLR	BGM	MLR	CHE	CMB	MAD	BHO	LKW	DEL
Total number recruited	998	1,000	1,000	1,000	1,003	1,000	1,000	1,000	1,001	998	1,000	997
Number of seeds	2	2	2	2	2	2	2	2	2	2	2	3
Recruitment time (days)	118	97	104	121	87	81	100	124	70	80	96	157
Number of waves (by seed)	22 (22, 17)	17 (17, 2)	13 (12, 13)	16 (16,16)	28 (13, 28)	21 (11, 21)	14 (11, 14)	20 (18, 20)	11 (11, 11)	23 (21, 23)	25 (25, 21)	21 (18, 21, 8)
Number of recruits by seed	619, 379	997, 3	291, 709	126, 874	73, 930	73, 927	341, 659	451, 549	475, 526	412, 586	465, 535	266, 544, 187
Coupon response rate (%)	52	51	50	51	51	50	50	52	50	69	50	50
Median network size*	10	20	60	5	4	18	8	15	10	5	4	10
Prevalence of unaware HIV-infected persons (%)	13.7	8.4	4.3	5.3	3.7	1.8	0.5	10.5	2.3	4.0	6.0	4.0
Rate of detection of unaware HIV-infected persons per day	1.16	0.87	0.41	0.44	0.43	0.22	0.05	0.85	0.33	0.50	0.38	0.42
Cost per unaware HIV-infected person identified (USD)	200.7	300.5	424.0	455.5	430.1	1,330.4	5,636.5	189.0	914.3	458.6	482.8	767.1
Prevalence of HIV viremia (%)	13.2	10.5	4.9	6.4	4.4	1.8	2.4	9.0	4.8	5.1	6.0	3.6
Rate of detection of HIV viremic persons per day	1.12	1.08	0.47	0.53	0.51	0.22	0.24	0.73	0.69	0.64	0.38	0.38
Cost per viremic person identified (USD)	304.7	328.1	533.7	473.5	454.0	1,405.6	1,360.4	340.0	622.8	440.7	574.2	927.5

*Number of MSM seen in prior 30 days.

BGM, Belgaum; BHO, Bhopal; BLR, Bengaluru; CHE, Chennai; CMB, Coimbatore; DEL, New Delhi; HYD, Hyderabad; LKW, Lucknow; MAD, Madurai; MLR, Mangalore; MSM, men who have sex with men; VJW, Vijayawada; VZG, Vishakhapatnam; USD, US dollars.

The median detection rate of HCV-infected individuals who were unaware of their status across all sites combined was 2.5 per day, but the rate was as high as 10.2 per day in Churachandpur (Table 3). The detection rate of HCV viremic PWID ranged from approximately 1 every 3 days in Gangtok to 9 per day in Churachandpur. Underlying HCV prevalence and prevalence of HCV viremia in the community were positively associated with a more rapid detection rate for HCV viremic PWID (linear regression coefficient per 1-percentage-point increase in prevalence of HCV and HCV viremia was 0.13 and 0.16, respectively; *p* = 0.004 for both).

### Cost per unaware and viremic person identified

**Fig 3:**
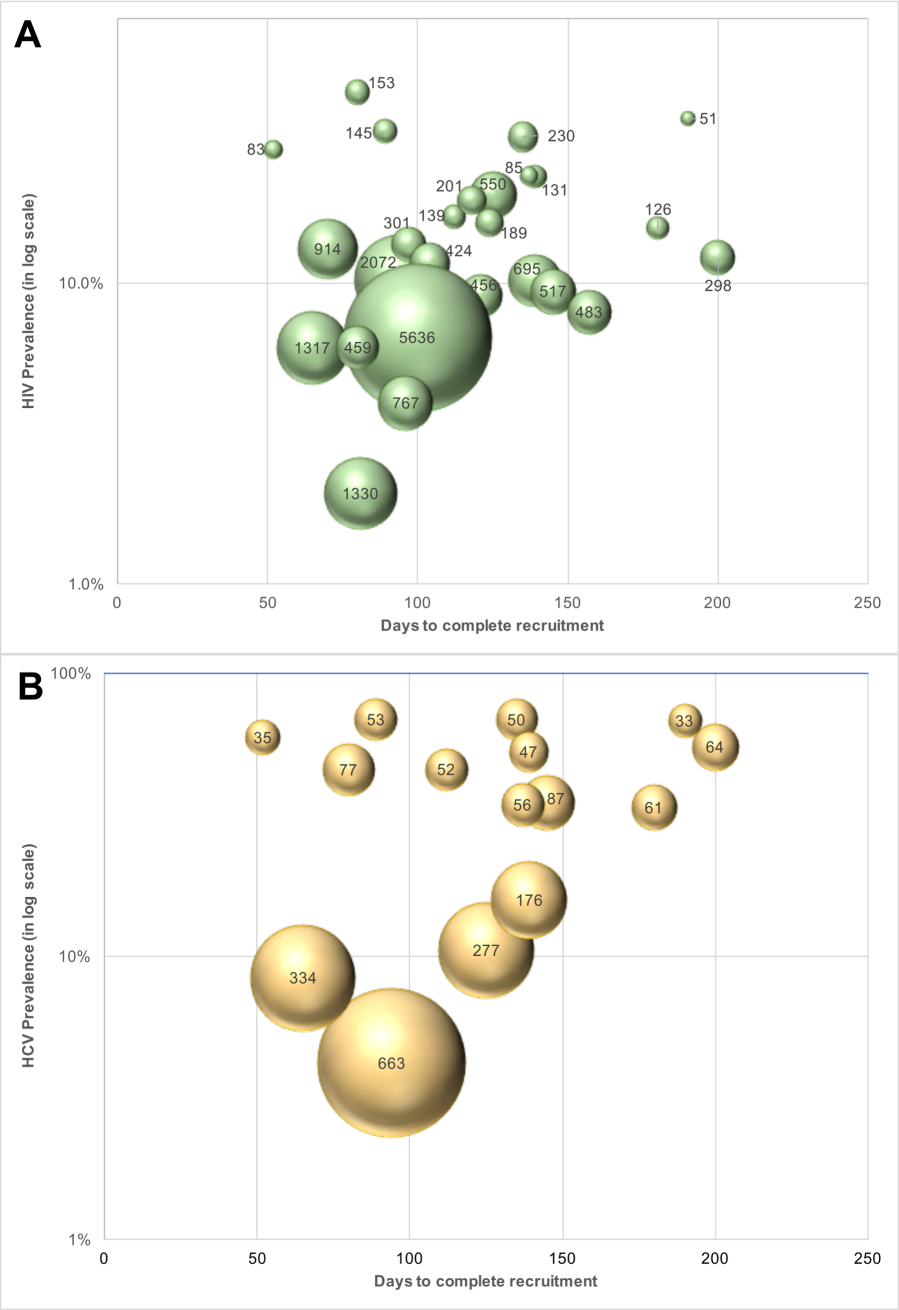
Cost of identifying unaware HIV- and HCV-infected MSM and PWID in India using respondent-driven sampling. (A) Cost of identifying an unaware HIV-infected person at each site (*n =* 27 sites; 15 PWID sites and 12 MSM sites). The size of the bubbles is proportional to the cost. (B) Cost of identifying an unaware HCV-infected person at each PWID site (*n =* 15). The size of the bubble is proportional to the cost. HCV, hepatitis C virus; MSM, men who have sex with men; PWID, people who inject drugs.

The cost of identifying 1 unaware HIV-infected individual ranged from US$51 to US$2,072 across PWID sites (Table 3) and from US$189 to US$5,637 across MSM sites (Table 4). The median cost of identifying an HIV viremic individual was US$367 (site range: US$123–US$1,406). At PWID sites, the cost ranged from US$123 to US$858, and the range extended from US$305 to US$1,406 at the MSM sites. The cost of identifying viremic individuals was strongly associated with underlying prevalence of viremia in the community (linear regression coefficient per 1-percentage-point increase in prevalence of viremia: −US$31.8; *p* < 0.001; Fig 3).

Incorporating all site operational and RDS costs without taking into account HIV antibody and HIV RNA testing costs, the median site cost per unaware HCV-infected PWID identified was US$50 (site range: US$27–US$549). The median site cost to identify 1 HCV viremic individual was US$194 (range: US$158–US$723). The cost of identifying 1 unaware HCV-infected individual was associated with underlying community HCV prevalence (linear regression coefficient per 5-percentage-point increase in HCV prevalence: −US$29.9; *p* < 0.001; Fig 3). If HCV testing is incorporated into an existing RDS program framework (as in this study for HIV), the additional cost to identify 1 unaware HCV-infected PWID is US$11 (site range: US$7–US$150).

## Discussion

In this study, RDS was able to identify a substantial number of HIV-infected and HCV-infected individuals in key populations who were unaware of their infection and out of care. Moreover, the deeper RDS penetrated within a community, the more likely it was to identify individuals with HIV and HCV viremia, the groups at the highest risk for transmission. Costs were relatively low, particularly in settings with high disease burden. RDS was also able to reach communities across the city via a single site. Combining RDS (or other network-driven recruitment approaches) with strategies focused on linkage to care may be a viable option for achieving the 90-90-90 targets in key populations in resource-limited settings.

RDS was originally conceived as an HIV prevention strategy and as a means to arrive at representative estimates of disease burden in hidden populations, populations with no obvious sampling frame [13,14]. Consequently, RDS is most commonly used in surveillance studies among MSM and PWID and has become the cornerstone for accrual of samples of MSM and PWID globally. The majority of RDS samples comprise ~400–600 participants, and samples are typically terminated either when the sample achieves equilibrium or the desired sample size has been accrued. However, in recent years, RDS has been incorporated into trials particularly among MSM, PWID, and female sex workers to recruit the study population [20], identify high-risk individuals [15,16], or measure the impact of an intervention on the community [21,22]. Further, data from a study among 722 MSM in Nigeria suggested that deeper RDS waves were associated with lower likelihood of awareness of HIV status and being on ART [23]. Currently, there is a trial in progress (HPTN 078) that is assessing the ability of “deep-chain” RDS (defined as recruitment beyond the sixth wave) to identify viremic MSM in the US [24], reflecting that the potential for RDS to be used as an intervention is increasingly being recognized.

The data in this report represent one of the largest samples, to our knowledge, of MSM and PWID systematically collected using RDS across diverse settings at different epidemic stages within the same country. Our findings have implications for potential tailoring of RDS as an intervention in these 2 key populations. It has been hypothesized that the deeper RDS penetrates within a community (measured by the number of waves), the more likely it is to recruit hidden populations [13]. These distal recruits are also hypothesized to have relatively smaller networks (i.e., to be less connected) compared to those recruited in the early waves. Indeed, this was supported in our data by ethnographic work at the sites, which suggested that more participants recruited in later waves had never before visited the local non-governmental organization through which the RDS was operating, and demonstrated that those recruited in later waves were significantly less likely to be engaged in HIV and HCV services. This was particularly apparent for HCV, for which testing but not treatment services were available in India at the time of this study. For HCV, people who were recruited in earlier waves were significantly more likely to be aware of their HCV status, suggesting that those who are more connected within their community have more access to testing services, but there was no difference in HCV viremia by wave, which may reflect the near absence of treatment availability at the time of this study. By contrast, for HIV, both diagnostic and treatment services are widely available, and the earlier recruits were more likely to be aware of their status and virologically suppressed compared to later recruits, suggesting that the deeper the RDS runs, the more likely it will be to identify persons at highest risk for transmission of HIV. This has also been demonstrated among MSM in Nigeria [23].

It is important to note that there was extensive variability in the efficiency of RDS in identifying unaware and viremic persons by site. At sites with low HIV prevalence, the yield tended to be lower and the costs significantly higher (e.g., MSM in Chennai and Mangalore and PWID in Gangtok and Bhubaneswar). By contrast, among PWID in Churachandpur and Imphal, more than 2 viremic persons were being detected each day the RDS was operational. Among MSM sites in general, the yield was lower relative to PWID, likely attributable to the lower HIV prevalence of HIV in MSM compared to PWID [18,19]. Yet, it is important to note that even in MSM populations in settings such as Hyderabad and Vijayawada in the high-prevalence state of Andhra Pradesh, at least 1 individual with HIV viremia was detected each day RDS was ongoing.

We observed similar variability with respect to the costs per unaware or viremic individual identified. Sites with high disease prevalence had low costs and sites with low disease burden had higher costs, and this was consistent both at MSM and PWID sites. A recent cost-effectiveness analysis suggested that screening all persons in India for HIV once every 5 years compared to the current standard of testing (at presentation with AIDS-defining illness or at presentation to an integrated counseling and testing center, antenatal center, or sexually transmitted infection clinic) would be “cost-effective,” and a one-time screening of the national population would be “very cost-effective” as per WHO criteria for cost-effectiveness [25]. The prevalence of HIV in India is 0.26% [26]; using this prevalence and the market value of US$3.1 for an HIV test, it would cost US$1,192 to find 1 infection in the national population, without accounting for staffing, infrastructure, community mobilization, etc. Yet this approach was deemed to be “very cost-effective.” While we do not present a cost-effectiveness model in this paper, the cost of identifying 1 person with HIV viremia was lower than this threshold at 25 out of the 27 sites, highlighting the potential cost-effectiveness of combining RDS with other strategies to improve linkage to care and viral suppression. Further, cost-effectiveness would be significantly improved by incorporating screening for other diseases that impact mortality and disease transmission (e.g., tuberculosis and HCV in PWID, and syphilis and HSV-2 in MSM). The additional cost of identifying 1 HCV-infected PWID with viremia across the 15 PWID sites was only US$136 (and this includes market value for HCV antibody and HCV RNA testing, which amount to US$105). Given the low price of HCV therapy in India (~US$300 per 12-week regimen of sofosbuvir and daclatasvir) and the potential to prevent cirrhosis and hepatocellular carcinoma, this could potentially be very cost-effective.

These data collectively highlight the potential of RDS (if combined with other linkage-to-care strategies) to reach individuals who could benefit from treatment of HIV and are at highest risk for transmission. Yet questions remain on how best to optimize RDS. One might argue that running RDS indefinitely at a site may provide the highest yield. An alternate argument could be to continue RDS until certain programmatic targets have been achieved (e.g., recruit 500 HIV viremic PWID). Likely, these targets would need to be tailored to the setting based on underlying population size, disease prevalence, and the proportion of infected individuals with viral suppression. As a compromise, RDS could be allowed to run until sample proportions have stabilized (e.g., equilibrium is achieved). Generally, this occurs around 300–400 persons, as was the case in all our 27 samples; HIV prevalence remained stable between this point and when the final sample size was achieved. The decision to continue the RDS or not could be based on the HIV prevalence within a site sample once equilibrium is attained. As clearly highlighted in the data, settings with historic epidemics and good access to ART such as Chennai will not be good candidate sites for RDS or other network-driven recruitment approaches to find viremic persons. In contrast, emerging sites with limited diagnostic and treatment access such as Kanpur are potentially good candidate sites for such an approach. The optimal number of coupons handed out is also worth further consideration. In this study, all participants were provided with 2 coupons as RDS was being used for surveillance, and depth was critical for generalizability of the sample. However, if RDS was being used to find unaware and viremic individuals, providing more coupons could result in identification of cases more rapidly, but at the same time the site would need to be suitably staffed to handle an increased client load. Alternatively, more coupons could be provided to select participants with characteristics suggestive of being “good” recruiters—participants more likely to hand out coupons to unaware and viremic persons. Future studies will need to explore these areas to optimize the efficiency of RDS to identify unaware and viremic persons.

A number of limitations need to be acknowledged. This study was conducted exclusively in India and may not be generalizable to other settings. However, we did have data on a range of epidemic stages across diverse geographic settings within India. It is challenging to compare the costs of this RDS-based strategy with those of the standard of care (targeted interventions) in India because these costs are not widely available. Further, while we used biometric data to exclude duplicates in our setting, this is not the standard across other targeted interventions in India. Our study was unique in that we recruited such large samples with only 2 seeds. This is in contrast to most studies, which often use upwards of 6 to 12 seeds. It is possible that our networks were more interconnected than others or that there was a large number of eligible individuals within the target area. It is possible that RDS is only effective in settings where the population is one large interconnected network. However, in other samples that have required more seeds (e.g., 7 in the study among MSM in Nigeria) [23], the same association between sample depth and proportion unaware of their HIV status has been seen. Participants were classified as unaware of their HIV and HCV status based on self-report, which is subject to bias/misclassification. Ideally, verification of prior diagnoses would be conducted with clinical records or electronic medical records. However, the availability of HIV RNA and HCV RNA data provided an objective outcome for the analyses, and these data are possibly the most important data if control or elimination is to be achieved.

In conclusion, in this large study among MSM and PWID across diverse settings in India, RDS was effective in reaching a large sample of unaware and viremic HIV-infected and HCV-infected individuals at relatively low cost, reflecting its potential for use as an intervention to improve detection of individuals with the potential to transmit HIV and/or HCV infection. In LMIC settings with high disease burden among key populations, combining RDS (or other network-driven recruitment approaches) with strategies focused on linkage to care may be a viable option for achieving the 90-90-90 targets.

## Supporting information

S1 Fig Flow diagram of study population. (PDF)

S1 Text STROBE-RDS checklist. (PDF)

S2 Text Institutional review board approvals. (PDF)

## Abbreviations

HAART: highly active antiretroviral therapy
HCV: hepatitis C virus
INR: Indian rupees
LMICs: low- and middle-income countries
MSM: men who have sex with men
PWID: people who inject drugs
RDS: respondent-driven sampling
UNAIDS: Joint United Nations Programme on HIV/AIDS
