# Supplementary material for: Respondent-driven sampling for identification of HIV- and HCV-infected people who inject drugs and men who have sex with men in India: A cross-sectional, community-based analysis
Source: PLoS Med. 2017 Nov 28;14(11):e1002460. doi: 10.1371/journal.pmed.1002460 (PMC5705124; doi:10.1371/journal.pmed.1002460)

S1 Fig. Flow diagram of study population

(A) People who inject drugs across 15 cities

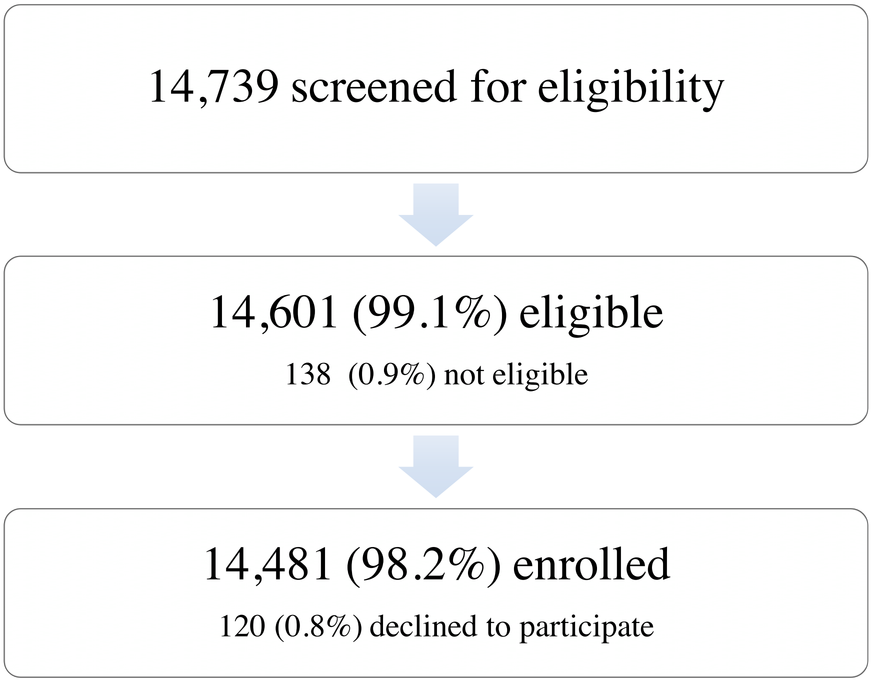

(B) Men who have sex with men across 12 cities

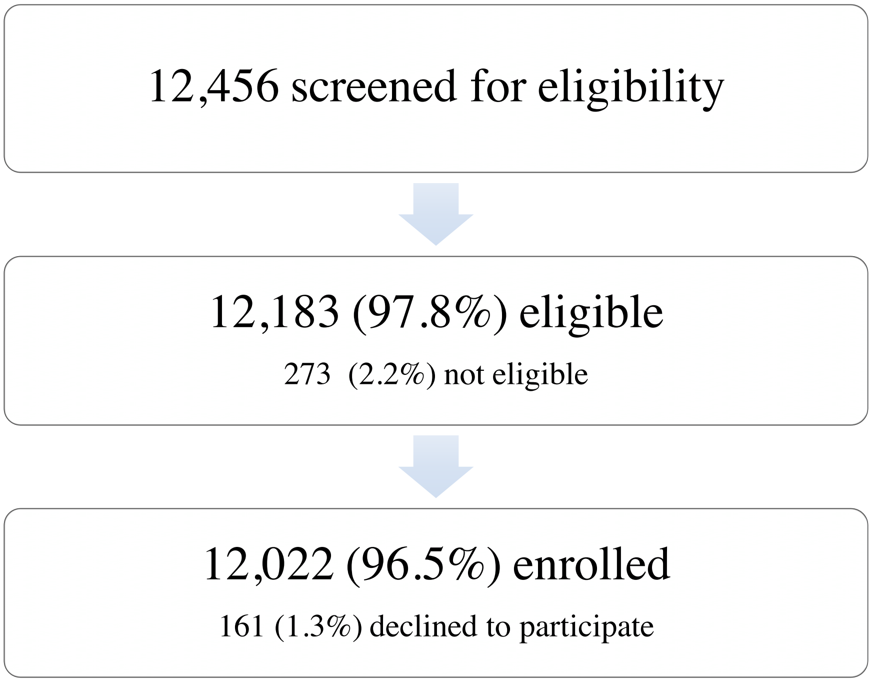

Supplement: S1 Fig — (PDF) [file pmed.1002460.s001.pdf]
