# Supplementary material for: Respondent-driven sampling for identification of HIV- and HCV-infected people who inject drugs and men who have sex with men in India: A cross-sectional, community-based analysis
Source: PLoS Med. 2017 Nov 28;14(11):e1002460. doi: 10.1371/journal.pmed.1002460 (PMC5705124; doi:10.1371/journal.pmed.1002460)
Supplement: S1 Text — (PDF) [file pmed.1002460.s002.pdf]

Item	#	STROBE-RDS recommendation[1]	Comment and/or section in the manuscript
**Title and Abstract**	1	(a) Indicate “respondent-driven sampling" in the title or abstract	Respondent-driven sampling in title.
		(b) Provide in the abstract an informative and balanced summary of what was done and what was found	Abstract includes summary of study and findings.
**Introduction**			
Background/ rationale	2	Explain the scientific background and rationale for the investigation being reported	Introduction
Objectives	3	State specific objectives, including any pre-specified hypotheses	Introduction, last paragraph.
**Methods**			
Study design	4	(a) Present key elements of study design early in the paper	Methods, Study Setting
		(b) State why RDS was chosen as the sampling method	Methods, Study Setting
Setting	5	a) Describe the setting, locations, and relevant dates, including periods of recruitment, and data collection	Methods, Study Setting
		(b) Describe formative research findings used to inform RDS study	Methods, Study Population

Item	#	STROBE-RDS recommendation[1]	Comment and/or section in the manuscript
Participants	6	(a) Give the eligibility criteria, and the sources and methods of selection of participants. Describe how participants were trained/ instructed to recruit others, number of coupons issued per person, any time limits for referral	Methods, Study Population
		(b) Describe methods of seed selection and state number at start of study and number added later	Methods, Study Population, first paragraph
		(c) State if there was any variation in study procedures during data collection (e.g., changing numbers of coupons per recruiter, interruptions in sampling, or stopping recruitment chains)	Results, RDS Summary Metrics - recruitment in Moreh was terminated early due to civil unrest.
		(d) Report wording of personal network size question(s)	Methods, Statistical Methods, second paragraph
		(e) Describe incentives for participation and recruitment	Methods, Study Population, first paragraph
Variables	7	(a) If applicable, clearly define all outcomes, correlates, predictors, potential confounders, effect modifiers, and diagnostic criteria	Methods, Study Procedures and Statistical Methods

Item	#	STROBE-RDS recommendation[1]	Comment and/or section in the manuscript
		(b) State how recruiter-recruit relationship was tracked	Methods, Study Population, first paragraph
Data sources/ measurement	8	(a) For each variable of interest, give sources of data and details of methods of measurement. Describe comparability of measurement methods if there is more than one group	Methods, Statistical Methods
		(b) Describe methods to assess eligibility and reduce repeat enrollment (e.g. coupon manager software, biometrics)	Methods, Study Population
Bias	9	Describe any efforts to address potential sources of bias	Inclusion of objective outcome measures in addition to self-reported outcomes; standardized interviewer training and electronic data capture with field restrictions and logic checks
Study size	10	Explain how the study size was arrived at	Results, RDS Summary Metrics, first paragraph
Quantitative variables	11	Explain how quantitative variables were handled in the analyses. If applicable, describe which groupings were chosen, and why	Methods, Statistical Methods

Item	#	STROBE-RDS recommendation[1]	Comment and/or section in the manuscript
Statistical methods	12	(a) Describe all statistical methods, including those to account for sampling strategy (e.g. the estimator used) and, if applicable, those used to control for confounding	Methods, Statistical Methods, second paragraph
		(b) State data analysis software, version number and specific analysis settings used	Methods, Statistical Methods
		(c) Describe any methods used to examine subgroups and interactions	Methods, Statistical Methods
		(d) Explain how missing data were addressed	Methods, Statistical Methods
		(e) Describe any sensitivity analyses	Not applicable
		(f) Report any criteria used to support statements on whether estimator conditions or assumptions were appropriate	We have presented findings related to estimator conditions/assumptions in the baseline papers previously published [2,3].
		(g) Explain how seeds were handled in analysis	Methods, Statistical Methods
**Results**			
Participants	13	a) Report the numbers of individuals at each stage of the study —e.g., numbers potentially eligible,	Supplementary Figure 1

Item	#	STROBE-RDS recommendation[1]	Comment and/or section in the manuscript
		examined for eligibility, confirmed eligible, included in the study, and analyzed	
		(b) Give reasons for non-participation at each stage (e.g., not eligible, does not consent, decline to recruit others)	Supplementary Figure 1
		(c) Consider use of a flow diagram	Supplementary Figure 1
		(d) Report number of coupons issued and returned	Table 3 and 4
		(e) Report number of recruits by seed and number of RDS recruitment waves for each seed. Consider showing graph of entire recruitment network	Table 3 and 4
		(f) Report recruitment challenges (e.g. commercial exchange of coupons, imposters, duplicate recruits) and how addressed)	No significant challenges
		(g) Consider reporting estimated design effect for outcomes of interest	Not applicable
Descriptive data	14	a) Give characteristics of study participants (e.g., demographic, clinical, social) and, if applicable,	Table 1

Item	#	STROBE-RDS recommendation[1]	Comment and/or section in the manuscript
		information on correlates and potential confounders. Report unweighted sample size and percentages, estimated population proportions or means with estimated precision (e.g., 95% confidence interval)	
		(b) Indicate the number of participants with missing data for each variable of interest	Methods, Statistical Methods
Outcome data	15	If applicable, report number of outcome events or summary measures	Table 1
Main results	16	(a) Give unadjusted and study design adjusted estimates and, if applicable, confounder adjusted estimates and their precision (e.g., 95% confidence intervals). Make clear which confounders were adjusted for and why they were included	Table 1
		(b) Report category boundaries when continuous variables were categorised	Wave categories reported in Table 2.
		(c) If adjustment of primary outcome leads to marked changes, report information on factors influencing	Not applicable

Item	#	STROBE-RDS recommendation[1]	Comment and/or section in the manuscript
		the adjustments (e.g. personal network sizes, recruitment patterns by group, key confounders)	
Other analyses	17	Report other analyses done—e.g., analyses of subgroups and interactions, sensitivity analyses, different RDS estimators and definitions of personal network size	Not applicable
**Discussion**			
Key results	18	Summarise key results with reference to study objectives	Discussion, first paragraph
Limitations	19	Discuss limitations of the study, taking into account sources of potential bias or imprecision. Discuss both direction and magnitude of any potential bias	Discussion, second to last paragraph
Interpretation	20	Give a cautious overall interpretation of results considering objectives, limitations, multiplicity of analyses, results from similar studies, and other relevant evidence	Discussion
Generalisability	21	Discuss the generalisability (external validity) of the study results	Discussion, last paragraph

Item	#	STROBE-RDS recommendation[1]	Comment and/or section in the manuscript
**Other information**			
Funding	22	Give the source of funding and the role of the funders for the present study and, if applicable, for the original study on which the present article is based	Acknowledgments section

1: White RG, Hakim AJ, Salganik MJ, et al. Strengthening the Reporting of Observational Studies in Epidemiology for respondent-driven sampling studies: "STROBE-RDS" statement. Journal of Clinical Epidemiology. 2015;68(12):1463-1471. doi:10.1016/j.jclinepi.2015.04.002.
2: Lucas GM, Solomon SS, Srikrishnan AK, Agrawal A, Iqbal S, Laeyendecker O, et al. High HIV burden among people who inject drugs in 15 Indian cities. *AIDS*. 2015;29(5):619-28.
3: Solomon SS, Mehta SH, Srikrishnan AK, Vasudevan CK, McFall AM, Balakrishnan P, et al. High HIV prevalence and incidence among MSM across 12 cities in India. *AIDS*. 2015;29(6):723-31.
