# Supplementary material for: Respondent-driven sampling for identification of HIV- and HCV-infected people who inject drugs and men who have sex with men in India: A cross-sectional, community-based analysis
Source: PLoS Med. 2017 Nov 28;14(11):e1002460. doi: 10.1371/journal.pmed.1002460 (PMC5705124; doi:10.1371/journal.pmed.1002460)
Supplement: S2 Text — (PDF) [file pmed.1002460.s003.pdf]

JOHNS HOPKINS MEDICINE

Office of Human Subjects Research
Institutional Review Boards

1620 McElderry Street, Reed Hall, Suite B-130
Baltimore, Maryland 21205-1911
410-955-3008
410-955-4367 Fax
e-mail: jhmirb@jhmi.edu

Date: January 6, 2017

CONTINUING REVIEW APPROVAL

**Review Type:**	Expedited
**Principal Investigator:**	Gregory Lucas
**Number:**	NA_00047702 / CR00013850
**Title:**	Integrated Care Clinics for IDUs in India: a Cluster Randomized Trial
**Committee Chair:**	Richard Moore
**IRB Committee:**	IRB-3

Date of approval: January 2, 2017

Date of Expiration: January 1, 2018

The JHM IRB approved the above-referenced Continuing Review.

Enrollment is active. Approval includes a supplemental table, DSMB recommendation.

If this study is a clinical trial and data collection is complete for the perspecified primary outcome, Section 801 of the Food and Drug Administration Amendments Act requires reporting of summary results information at http://www.clinicaltrials.gov. Reporting must be done within 12 months of completing data collection for the prespecified primary outcome, regardless of sponsor or funding source. Failure to comply with this law may result in civil penalties. For more information on results reporting go to http://www.clinicaltrials.gov. If the study is registered with Clinicaltrials.gov and is closed to recruitment and enrollment, the record must be updated within 30 days to reflect the study's enrollment status. See http://clinicaltrials.gov/ct2/manage-recs/how-edit for more information. Questions can be directed to register@clinicaltrials.gov.

Date of Approval and Expiration Date: The approval and expiration date for this research are listed above. If the approval lapses, the research must stop and you must submit a request to the IRB to determine whether it is in the best interests of individual participants to continue with protocol-related procedures.

Changes in Research: All proposed changes to the research must be submitted using a Change in Research application. The changes must be approved by the JHM IRB prior to implementation, with the following exception: changes made to eliminate apparent immediate hazards to participants may be made immediately, and promptly reported to the JHM IRB.

Continuing Review: Continuing Review Applications should be submitted at least 6 weeks prior to the study expiration date. Failure to allow sufficient time for review may result in a lapse of approval. If the Continuing Review Application is not submitted prior to the expiration date, your study will be terminated and a New Application must be submitted to reinitiate the research.

Unanticipated Problems: All unanticipated problems must be submitted using a Protocol Event Report.

If this research has a commercial sponsor, the research may not start until the sponsor and JHU have signed a contract.

The Johns Hopkins Institutions operates under multiple Federal-Wide Assurances: The Johns Hopkins University School of Medicine - FWA00005752, The Johns Hopkins University School of Nursing - FWA00006088, The Johns Hopkins Hospital and Johns Hopkins Health Systems - FWA00006087, Johns Hopkins Bayview Medical Center - FWA00006089, Howard County General Hospital - FWA00005743, Hugo W. Moser Research Institute at Kennedy Krieger, Inc. - FWA00005719, Johns Hopkins Community Physicians - FWA00002251, Suburban Hospital and Health System - FWA00005924

JOHNS HOPKINS
BLOOMBERG SCHOOL of PUBLIC HEALTH

Institutional Review Board Office

615 N. Wolfe Street / Room E1100
Baltimore, Maryland 21205-2179
Phone: 410-955-3193
Toll Free: 1-888-262-3242
Fax: 410-502-0584
Email: jhsph.irboffice@jhu.edu
Website: www.jhsph.edu/irb

FWA #00000287

CONTINUING REVIEW APPROVAL NOTICE

Date: June 21, 2017

To: David Celentano, ScD, and MHS
Department of Epidemiology

From: Joanne Katz, ScD
Chair, IRB-FC

Re: **Study Title**: "Preventing HIV Infection among MSM in India"
IRB No: 00002838
Study Expiration Date: June 20, 2018

The JHSPH IRB-FC reviewed and approved the Progress Report submitted for the above referenced study at its meeting on June 21, 2017. Approval of the study is valid for the period of June 21, 2017 to June 20, 2018.

This approval is inclusive of all previously approved documents. If you are actively enrolling, or if participants are in follow-up, you may only use previously approved consent forms and other study documents.

**Single Reviewer** ☐ **Convened** ☒<br><br>DHHS 46.110 . . ☐ DHHS...... ☒<br>FDA 56.110 . . . ☐ FDA......... ☐<br><br>Category:	**Consent/Parental Permission Required From:**<br>Adult Participant............. ☒<br>LAR .... ☐<br>One Parent .... ☐<br>Two Parents.................... ☐<br>Legal Guardian................. ☐<br>(Foster Care Children)	**Form of Consent/Permission:**<br>Written Consent........................ ☒<br>Waiver of Signature.................. ☐<br>(Oral Script)<br>Waiver of Informed Consent.... ☐<br>HIPAA Authorization................. ☐<br>HIPAA Waiver............................ ☐<br>No Longer Enrolling.................. ☐	**Study Site(s):**<br><br>U.S. ☐ International ☒<br><br>**List Country(ies):**<br><br>**India**
**GWAS** .... ☐			
**Vulnerable Populations:**<br><br>Children .... ☐<br>Foster Care Children .... ☐<br><br>**DHHS** **FDA**<br>46.404 . . . . ☐ 50.51........ ☐<br>46.405. . . . ☐ 50.52........ ☐<br>46.406 . . . . ☐ 50.53........ ☐	**Assent Required From:**<br>No children (waived) .. ... ☐<br>Children aged:__________ ☐<br><br>**Form of Assent:**<br>Written .... ☐<br>Oral .... ☐<br>Assent Statement in Parent Permission .... ☐	**Pregnant Women/Fetuses**<br>46.204............................ ☐<br><br>**Neonates**<br>46.205 .... ☐<br><br>**Prisoners**<br>46.305 .... ☐<br>46.306 .... ☐<br>Epidemiological Research... ☐	**Sample Size:**<br>(screened plus enrolled)<br><br>**22830**<br><br>**Secondary Data Analysis**:<br>(# specimens/participants)

As principal investigator of the study, you are responsible for fulfilling the following requirements of approval:

1) The co-investigators listed on the application should be kept informed of the status of the research.

2) Submit an Amendment Request Form for any changes in research. These changes in research are required to be reviewed and approved prior to the activation of the changes, with the following exception: changes made to eliminate an apparent immediate hazard to the research participant may be instituted immediately and the JHSPH IRB should be informed of such changes promptly.

3) Unanticipated problems involving risks to participants or others must be reported to the JHSPH IRB in accordance with the **JHSPH IRB Organizational Policy on Reports of Unanticipated Problems Involving Risks to Participants or Others.** An Adverse Event Form must be submitted to the IRB immediately.

4) Only consent forms with a valid JHSPH IRB approval stamp may be presented to participants, unless otherwise approved by the IRB. All consent forms signed by participants enrolled in the study should be retained on file. The Office of Graduate Education and Research conducts periodic compliance monitoring of protocol records, and consent documentation is part of such monitoring.

   Federal regulations require review of approved research not less than once per year. **Please submit a progress report <u>no later than 6 weeks</u> before the approval lapse date. We recommend that YOU USE YOUR OUTLOOK CALENDAR, OR OTHER ELECTRONIC REMINDER CALENDAR TOOL, to set a timely reminder notification for this submission to avoid a lapse in approval.** This will allow sufficient time for review of the Progress Report to be completed prior to the expiration date. Failure to submit a Progress Report for continuing review prior to the expiration date will result in termination of the research, at which point new participants may not be enrolled and currently enrolled participants must discontinue participation in the study. All ongoing research activities must stop immediately, including data analysis.

5) If your research involves international travel, please don't forget to register with the International Travel Registry https://apps4.jhsph.edu/ITR/Default.aspx so that the School may locate you in the event of an emergency.

JK/rch

Institutional Review Board

(IRB 00001423/FWA 00000672)

Y.R.G.CARE
www.yrgcare.org

VHS-YRG CARE MEDICAL CENTRE
VOLUNTARY HEALTH SERVICES
TARAMANI, CHENNAI – 600 113
INDIA

Tel. : +91- 44- 22542929
Fax : +91- 44- 22542939
email : info@yrgcare.org

IRB CHAIRPERSON

Dr. Jayakar Paul
jayakarsaj@hotmail.com

IRB CO-CHAIRPERSON

Dr. V. Kumaraswami
kumaraswami@gmail.com

MEMBERS

Mr.Aravind Subramaniam
maravindsubraman@hotmail.com

Mr. K. Arumugam
arumugam.2006@rediffmail.com

Mr. Benjamin Franklin
jfredrickf@yahoo.com

Dr.Jayapaul Azariah
Jazariah@yahoo.com

Dr. Jyotirmay Biswas
drjb@sankaranethralaya.org

Dr. P. Kuganantham
drkugan@yahoo.com

Dr.Malathy Duraisamy
mdurai@iitm.ac.in

Dr. A. Nalini
nalinianna@yahoo.com

Dr. T. Rajkumar
drtrajkumar@gmail.com

Dr. R. Ramakrishnan
drramakrishnan.nie@gmail.com

Dr. Saradha Suresh
drsaradhasuresh@gmail.com

Ms. Sarah Chanda
044 – 26747611

Dr. S. Shantha
thayshan@hotmail.com

Ms. Sheila Jayaprakash
sheilajayaprakash@yahoo.com

Dr. Suresh Kumar
msuresh@vsnl.com

IRB MANAGER

Dr. S. Swarnalakshmi
swarna@yrgcare.org

Date: March 9, 2011

To: Aylur K. Srikrishnan (Study PI), Research Manager, YRG CARE

Principal Investigators:
Dr. Gregory M. Lucas (Johns Hopkins University, USA)
Dr. Shruti h. Mehta (Johns Hopkins University, USA)
Dr. M. Suresh Kumar (Consultant, YRGCARE, INDIA)

Co-investigators:
Dr. David D. Celentano (Johns Hopkins University, USA)
Dr. Sunil Suhas Solomon (YRGCARE, India)
Dr. Suniti Solomon (YRGCARE, India)

Name of Project: Integrated Care Clinics for IDUs in India: A Cluster Randomized Trial,
Protocol Version Date February 16th, 2011

Ref.: Your Submission letter dated February 16, 2011

With reference to the above-mentioned project, the following document was submitted to the YRG CARE IRB for review and approval:

- Protocol, Version Date February 16th, 2011

The above-mentioned Protocol was reviewed by the Institutional Review Board of YRG CARE in its meeting held on March 5, 2011.

The IRB has approved and accepted the Protocol and will file appropriate documentation in the study file.

The IRB approval of the above-mentioned Protocol is granted with effect from March 5, 2011, and is valid till March 4, 2012. The Protocol will be submitted for re-approval in the Annual Review for the study to take place during the period February 5, 2011 – March 4, 2012.

I have signed in approval on behalf of the Institutional Review Board of YRG CARE.

Sincerely,

YRG FOUNDATION
IRB APPROVED
Signature of Co-Chair/Date 9/3/11

Dr. V. Kumaraswami
IRB Co-Chairperson

Institutional Review Board

(IRB 00001423/FWA 00000672)

Y.R.G.CARE
www.yrgcare.org

YRG CARE
VHS-YRG CARE MEDICAL CENTRE
VOLUNTARY HEALTH SERVICES
TARAMANI, CHENNAI – 600 113
INDIA

Tel. : +91- 44-39106600
Fax : +91- 44-22542939
email : info@yrgcare.org

IRB CHAIRPERSON
Dr. C.N. Paramasivan
Microbiologist (Scientist)

IRB CO-CHAIRPERSON
Dr. Saradha Suresh
Pediatrician (Scientist)

MEMBERS
Mr. Aravind Subramaniam
Advocate (Non-Scientist)

Mr. K. Arumugam
Lay person - Community (Non-Scientist)

Mr. Benjamin Franklin
Social Work (Non-Scientist)

Dr. Jyotirmay Biswas
Ophthalmic Pathologist (Scientist)

Dr. P. Kuganantham
Infectious Diseases/
General Medicine (Scientist)

Dr. Malathy Duraisamy
Economist (Non-Scientist)

Dr. K. G. Murugavel
Medical/ Molecular Virology (Scientist)

Dr. A. Nalini
Pathologist & Bioethicist (Scientist)

Dr. R. Ramakrishnan
Statistician (Non-Scientist)

Ms. Sheila Jayaprakash
Advocate (Non-Scientist)

Dr. C. B. Tharani
Clinical Pharmacologist (Scientist)

IRB MANAGER/MEMBER SECRETARY
Dr. S. Swarnalakshmi
Bioethicist (Non-Scientist)

ALTERNATE MEMBERS
Dr. Jayakar Paul
General Medicine (Scientist)

Dr. T. Rajkumar
Medicine (Scientist)

Dr. S. Shantha
Microbiologist (Scientist)

Dr. M. Suresh Kumar
Psychiatrist (Scientist)

ALTERNATE MEMBER SECRETARY
Ms. Rochelle D'Souza Yepthomi
Social Worker/Counselor (Non-Scientist

Date: April 15, 2017

To: Mr. A. K. Srikrishnan, Research Manager, YRG CARE, VHS, Chennai 600113;
Dr. David D Celentano (PI), ScD, MHS (JHBSPH)

CC: *Co-Investigators:*
Dr. Shruti H Mehta, JHBSPH, US
Dr. Frangiscos Sifakis, JHBSPH, US
Dr. Sunil Suhas Solomon, YRG CARE/, JHSPH, US

Name of Project: YRG CARE IRB Project No.: YRG - 160
Preventing HIV Infection among MSM in India,
Version No. 5.0, Version Date: March 14, 2014

Ref.: IRB Submission Form dated March 31, 2017

With reference to the above-mentioned project, the following documents were submitted to the YRG CARE IRB for review and approval:

- Continuing /Annual Review Application Form , Dated March 31, 2017
- Copy of Research Plan, Version 5.0, March 14, 2014

The above-mentioned documents were reviewed by the Institutional Review Board of YRG CARE in its meeting held on April 1, 2017. The IRB has approved and accepted the above documents and will file appropriate documentation in the study file.

The IRB re-approval of the above-mentioned Research Plan is granted with effect from April 1, 2017, and is valid till March 31, 2018. The same will be submitted for re-approval in the Continuing / Annual Review for the study to take place during the period March 1, 2018 – March 31, 2018.

I have signed in approval on behalf of the Institutional Review Board of YRG CARE.

Sincerely,

YRG FOUNDATION
IRB APPROVED
Signature of Chair/Date 15/4/17

Dr. C. N. Paramasivan
IRB Chairperson
